# Ten quick tips for biocuration

**DOI:** 10.1371/journal.pcbi.1006906

**Published:** 2019-05-02

**Authors:** Y. Amy Tang, Klemens Pichler, Anja Füllgrabe, Jane Lomax, James Malone, Monica C. Munoz-Torres, Drashtti V. Vasant, Eleanor Williams, Melissa Haendel

**Affiliations:** 1 Genestack Limited, Cambridge, Cambridgeshire, United Kingdom; 2 European Molecular Biology Laboratory, European Bioinformatics Institute (EMBL-EBI), Hinxton, Cambridgeshire, United Kingdom; 3 SciBite Limited, BioData Innovation Centre, Hinxton, Cambridgeshire, United Kingdom; 4 Phoenix Bioinformatics, Fremont, California, United States of America; 5 Bayer Business Services GmbH, BP Research and Development, Translational Sciences, Berlin, Germany; 6 Centre for Gene Regulation and Expression, School of Life Sciences, University of Dundee, Dundee, United Kingdom; 7 Genomics England, Queen Mary University of London, London, United Kingdom; 8 Linus Pauling Institute, Oregon State University, Corvallis, Oregon, United States of America; University of Toronto, CANADA

This is a *PLOS Computational Biology* Education paper.

## Introduction

Biomedical research is becoming more interdisciplinary, and data sets are becoming increasingly large and complex. Many current methods in research (omics-type analyses) generate large quantities of data, and the internet generally makes access easy. However, ensuring quality and standardisation of data usually requires more effort. Moreover, heterogeneity of data and the rate at which they are produced make it difficult to develop novel analytics or maximise the impact of data on scientific or clinical decision-making.

Shortcomings in data utility, reusability, and quality can be addressed by biocuration, which is the process of identifying, organising, correcting, annotating, standardising, and enriching biological data [1]. The primary aim of biocuration is to extract valuable knowledge from the corpus of available biological data and to accurately represent it in a computable manner so that it may be easy to understand and disseminate. Such knowledge includes experimental data on genes and proteins (both structural and functional) and on biological concepts like pathways, as well as experimental metadata, which provides important context for data interpretation and integrative analytics.

Biocuration is not a niche activity limited to professional biocurators contributing to public databases or the literature. Researchers today often use large data sets created in several labs or reuse other people’s public data, which are often not consistently annotated and are therefore in need of curation. There is even a ‘Research Parasite’ Award [2] for determining the most effective reuse of data. Ultimately, the responsibility of stewarding the scientific community’s data is shared by researchers, authors, biocurators, bioinformaticians, database managers, publishers, funders, data integrators, and end users. Of these, professionals with an interest in biocuration have come together and established the International Society for Biocuration (https://www.biocuration.org/). Despite collective international efforts, biocuration remains a challenge for everyone faced with the task of harmonising heterogeneous data. Many researchers may not even realise that they are performing biocuration on the data they are working with. We therefore created this guide to share our experience in curating private research data, public databases, and for large-scale data integration efforts, involving both published literature and primary data. The selected tips are aimed at providing practical guidelines to everyone wishing to structure biological data, from wet-lab researchers and computational biologists to early-career professional biocurators.

## Guidelines

### Tip 1. Know the subject area and assemble a team of experts

In biocuration, just like for any other aspect of research, domain knowledge is a prerequisite. The required level of knowledge depends on which specific curation tasks are carried out, and this in turn is linked to the use case of the curated data (see Tip 2). With roles in research spanning a wide spectrum, from wet-lab biologists to software developers, there will often be no single person with all of the required knowledge, and everyone’s input will have to be pooled. For example, curating RNA sequencing (RNA-seq) data will require knowledge in the biological domain, as well as sequencing-data file formats. When curating collaboratively, a mixture of expertise in a team can even be advantageous in that decisions on ambiguous curatorial cases represent a consensus and can help inform the development of unbiased curation standards (see Tip 6).

### Tip 2. Clearly define the intended use of the curated data

Thinking about the purpose of curating data is a crucial step, as defining use cases affects many other aspects of curation. The intended use determines the types and relationships of information that should be captured (the data model). The data model in turn informs the curation guidelines necessary to maintain it (see Tip 6). The guidelines may specify particular workflows, which themselves generate metadata that need to be captured (provenance and attribution).

For practical purposes, the above can be addressed by considering three questions. First, what kind of biological knowledge is one trying to capture? Curating biological entities, such as functional domains in proteins, is conceptually different from curating metadata of experimental data sets. Second, at which point of the data stream should curation be carried out? For researchers curating their own data, curation can happen concurrently with data collection. For curated resources, curation mostly happens post–data publication with access to literature, whereas for primary archives, this largely happens prior to publication. Third, what/who are the consumers of curated data? In modern research, the consumers are likely to be researchers mining and reusing data in the public domain, as well as computer programmes pulling curated data as an input for analysis pipelines.

The data models should be well documented, harmonised with community standards when possible (see Tip 6), and shared alongside the data. Furthermore, it is important to recognise that quality curated data will often be reused in ways that the provider may not foresee—for example, in large-scale data integration projects such as the disease mechanism discovery platform The Monarch Initiative [3]—so be prepared to reevaluate the known use cases periodically.

### Tip 3. Automate as much curation as possible

Automation can support curation efforts in many different ways. It can help speed up repetitive tasks, execute routine tasks, reduce error rates, and contribute to standardisation. For example, when curating cancer-related data, using a term from the National Cancer Institute’s Thesaurus (NCIt, http://purl.obolibrary.org/obo/ncit.owl) [4] would ensure standardisation at the point of data collection and allow other fields like normal cell origin or the cancer’s primary anatomic site to be automatically populated. Post–data collection, bespoke scripts can help automate collating disparate data in a common field in a standard template. Named entity recognition (NER) technologies can automate the extraction of information from free text, quickly short-listing relevant text for curators to review [5]. Automation also helps to trigger quality-control processes at certain points during curation or to run at scheduled times. This helps, for example, with normalising data in ‘date’ fields or with standardising terms for gender. Automation and standardisation are closely linked (see Tip 5). In the extreme, machine-learning or constraints-based procedures can annotate data sets with predictions (e.g., classifying tissue-staining images or suggesting a predicted name for a new protein sequence) independent of or with minimal curator input, making previously bare data sets more useful to users. It is paramount in these cases, however, to properly attribute and track data provenance (see Tip 6). Time saved by automation allows curators to focus on the edge cases requiring their expertise. Curators should identify which tasks are best suited for automation and—together with software developers or, indeed, on their own (see Tip 9)—come up with bespoke workflows.

### Tip 4. Share your data in a standard structure

Regardless of how data is persisted (see Tip 10), sharing data usually requires an exchange format (in most cases, files with a particular structure like a descriptive header and a body containing the actual data). For each subject area and data type, the curated data should be structured according to a metadata schema specifying mandatory fields. The structure should allow curated data to be exported in an agreed, standardised reporting format. One example is the data-reporting standards from the Clinical Data Interchange Standards Consortium (CDISC) (https://www.cdisc.org), which are an absolute requirement for those submitting clinical data to the United States Food and Drug Administration. Others include the Genome Reference Consortium’s (GRC) reporting standard for reference genome assemblies (https://www.ncbi.nlm.nih.gov/grc) and the Human Genome Organisation (HUGO) Gene Nomenclature Committee’s (HGNC) standard for human gene names [6]. Before starting a curation task, do a landscape review of what has been done to date to standardise similar data. This will require checking standards indices (for example, in FAIRsharing [https://www.fairsharing.org] [7]), searching the literature, and examining related databases.

Proposing a new ‘standard’ in a field in which well-established and accepted community standards already exist will cause confusion and lead to data interoperability issues. If you feel an existing standard needs improvement, consider contacting the standard’s maintainers and offer to contribute to its design instead. Occasionally, designing new standards is justified because existing standards do not fully describe the data.

### Tip 5. Use ontologies and persistent identifiers to annotate your data

We recommend the use of ontologies when describing biological concepts, as they provide a common vocabulary with persistent identifiers, as well as human- and machine-readable semantic relationships between concepts. The common vocabulary helps disambiguate language and avoid confusion. Perhaps the most well-known biological ontology is the Gene Ontology (GO) [8] [9], which describes gene and protein function and subcellular localisation. Curating your data using an ontology will impact many use cases for your data consumers, such as in enabling specific queries to be made or data from disparate sources to be merged based on common concepts.

When selecting an ontology, aim to use a well-established and stable one, even if it means extending it. Resources such as Bioportal (http://bioportal.bioontology.org) [10] or the Ontology Lookup Service (OLS) (https://www.ebi.ac.uk/ols) [11] provide an access point to browse and integrate biomedical ontologies. For some domains, like disease, there might be several competing ontologies available. The choice you make will depend on your use case and the degree of specificity and interoperability required [12]. If no ontology is available, even defining a small controlled vocabulary is better than just free text. Just be sure to publish it alongside your data and use persistent identifiers for your concepts (see below).

Unique persistent identifiers are key for ontological concepts, but they are provisioned for other entities as well. For example, digital object identifiers (DOIs) are provisioned for manuscripts and other content types. Persistence means that identifiers do not just vanish and that any changes are tracked throughout an identifier’s lifetime. If the associated concepts become obsolete, identifiers are retired and not reused. High-quality identifier provisioning and maintenance is key for downstream integration of data, provenance and attribution tracking, and reproducibility (see Tip 6 and [13]). In a nutshell, use identifiers wherever possible.

### Tip 6. Develop robust curation guidelines that include provenance and attribution

Whether you are an individual contributing to the community’s collective knowledge or part of a large curation team, biocuration is an inherently collaborative activity. Just like protocols help ensure consistent results in the laboratory, curation guidelines do the same for manual curation of data—they specify a set of steps and tools for a given task. Guidelines cannot replace curators’ domain knowledge but instead build on it. They also should be balanced so that they improve curation accuracy without impeding curation efficiency. Ideally, all curation procedures should be evaluated with multiple curators to ensure consistent application of curation guidelines. Measure intercurator consistency and evolve curation guidelines until consistency reaches a high level (e.g., > 90%–95%). Ontologies can help mitigate cases in which full annotation consistency cannot be achieved [14]. Curation guidelines should be fully documented, versioned, and made available to the users. This aids both curation consistency, transparency, and utility.

Curation guidelines should also describe the extent and method of documenting data provenance (i.e., where data is coming from and which transformations it has undergone) and of attributing steps in the curation process to particular agents (i.e., which person or process made which change). As recording every single step of curation is impractical, it is worth identifying key decisions in the curation workflow and recording in sufficient detail how such decisions are reached. An example of this is curation of variants for pathogenicity for a given disease using the tool ClinGen (https://www.clinicalgenome.org/curation-activities/variant-pathogenicity-curation/). In ClinGen, computable evidence and provenance are captured using the Scientific Evidence and Provenance Information Ontology (SEPIO, https://github.com/monarch-initiative/SEPIO-ontology) and the Evidence and Conclusion Ontology (ECO, http://www.evidenceontology.org). Such records would allow curators to reproduce the curatorial action should a new piece of similar data emerge and allow data users to fully understand why a particular data point was added.

### Tip 7. Curate early, stay cozy with the data

Metadata errors or omissions are much easier to rectify at the point of data collection or submission, when the research work is still fresh in the data depositor’s mind (provided good lab-book records have been kept [15]), as opposed to retrospective curation a few years down the line. Curating at the point of collection/submission can influence how and what data will be distributed and can also ensure that both data content and structure match curation goals. Data managers of a project are likely to have direct access to the data depositors, and so do curators in primary data archives where data deposition is often a prerequisite of journal publication. Curating early in the data stream also creates a shared sense of stewardship between the curator and the data provider. Through the dialogue, the curator may discover new information that can assist in making the data and their curation more impactful or more interoperable.

### Tip 8. Commit to maintaining data

Curated data should not and cannot be regarded as perfect, static, or the ‘final product’. Data need to be maintained, refreshed, and updated to maximise their potential in being reused and discovered. There are many scenarios in which recuration is required and beneficial to data consumers. For example, data capture or deposition standards may have been updated since the first curation, with additional mandatory metadata fields that hitherto did not exist and need to be retrofitted in previously curated data. New or updated ontologies that better align to the subject area of the curated data may have become available, and there may be a strong case for migrating existing annotations to new ontology terms to improve efficiency of data discovery. Updating ontology annotations does not alter the original data but means it can be kept up to date with the most current knowledge. Finally, curators’ or submitters’ mistakes may be revealed during the data reuse phase and need to be rectified.

### Tip 9. Learn basic programming for ad hoc data wrangling

Automation as discussed in Rule 3 often implies a well-designed workflow using appropriate (software) tools. Yet in many situations, curating data requires ad hoc solutions specific to the use case that may not yet be automatable by existing software. Examples might be drawn from cleaning up data, which often involves changing line endings or extracting particular columns from files, or exploratory analysis of large data sets. A curator who has basic scripting skills will be able to provide those solutions and carry on with the rest of the curation workflow without much delay. Being close to the data, a curator is in the best position to write a script that is fit for purpose and adapt it later if the requirements or the input change. Python is an example of a relatively easy-to-learn, general-purpose programming language. There are many freely available packages for bioinformatics (www.biopython.org) [16], data wrangling, plotting, and statistics.

### Tip 10. Persist the data and provide it in multiple formats

Commonly, curated data are stored in text files and tabulated spreadsheets. Although storing data in such forms may be useful in some cases, it is almost always better to persist the data and any curation that has been undertaken using a technology that offers long-term stability and versioning. This may be as simple as hosting the files on GitHub (https://github.com). Providing the data in a database with a documented schema instead of scattered across multiple spreadsheets is a bit more advanced but also more powerful, as data stored in a structured way in a database are easier to export to different formats. There are many different free database solutions available, covering many different use cases [17] [18]. Besides facilitating data sharing, centralised storage also aids backup procedures, which can be automated, ensuring that valuable curated data can be recovered should there be any technical problems (see also Tip 3).

An alternative to building your own database is to deposit your curated data in a suitable public database maintained by a major organisation, as recommended or required by some journals as a prerequisite of publication. For example, primary DNA sequence data should be deposited with the International Nucleotide Sequence Database Collaboration (INSDC, http://www.insdc.org/). Public databases maintained by major organisations are often more resourceful in persisting large volumes of data and adapting to the changing needs of the user community, such as with regard to supported formats of imported and exported data files (see Tip 4). It is worth noting that most public databases do not have resources to curate every submission, so presubmission curation is paramount in ensuring reusability of the deposited data.

As with most tasks, selecting a (technological) solution that is fit for purpose is key. Just as the data need to remain biologically relevant via recuration (see Tip 8), curation workflows likewise need to be refreshed with the latest data model (see Tip 2) and information technology to boost their chance of being persisted.

## Conclusion

With the ever-increasing amount, types, and complexity of data, biocuration is no longer a choice but a necessity to ensure that data are maximally useful. Biocurators are multitalented professionals with knowledge in biology, information management, and computing. The tips above provide a point of entry for researchers wanting to start contributing to biocuration as part of their projects or in their research area in general. Because of its diverse nature, curation offers great potential for expanding one’s skill set and may benefit careers. The recent introduction of a new postgraduate qualification in biocuration (https://www.ice.cam.ac.uk/course/postgraduate-certificate-biocuration) is one step in the right direction in recognising biocuration as a discipline on its own.

In the foreseeable future, however, securing adequate and stable funding remains one of the hurdles the biocuration community needs to overcome [1]. As funding bodies start mandating data management plans in research grant proposals—for example, by the Biotechnology and Biological Sciences Research Council (BBSRC) (https://bbsrc.ukri.org/funding/apply/application-guidance/data-management/) and the National Institutes of Health (NIH) (https://grants.nih.gov/grants/policy/data_sharing/data_sharing_guidance.htm)—we hope that the funding situation will ease somewhat, allowing biocuration to be woven into the data stream that emerges from the funded projects, and that the tips we have shared will lower the barrier to newcomers taking up this important task.
